# A Deletion in Exon 9 of the *LIPH* Gene Is Responsible for the Rex Hair Coat Phenotype in Rabbits (*Oryctolagus cuniculus*)

**DOI:** 10.1371/journal.pone.0019281

**Published:** 2011-04-28

**Authors:** Mathieu Diribarne, Xavier Mata, Céline Chantry-Darmon, Anne Vaiman, Gérard Auvinet, Stéphan Bouet, Séverine Deretz, Edmond-Paul Cribiu, Hubert de Rochambeau, Daniel Allain, Gérard Guérin

**Affiliations:** 1 INRA, UMR1313, Unité de Génétique Animale et Biologie Intégrative, INRA, Jouy-en-Josas, France; 2 INRA, UR967, Génétique Expérimentale en Productions Animales, INRA, Surgères, France; 3 INRA, UR631, Station d'Amélioration Génétique des Animaux, INRA, Castanet Tolosan, France; University of Texas M. D. Anderson Cancer Center, United States of America

## Abstract

The fur of common rabbits is constituted of 3 types of hair differing in length and diameter while that of rex animals is essentially made up of amazingly soft down-hair. Rex short hair coat phenotypes in rabbits were shown to be controlled by three distinct loci. We focused on the “r1” mutation which segregates at a simple autosomal-recessive locus in our rabbit strains. A positional candidate gene approach was used to identify the rex gene and the corresponding mutation. The gene was primo-localized within a 40 cM region on rabbit chromosome 14 by genome scanning families of 187 rabbits in an experimental mating scheme. Then, fine mapping refined the region to 0.5 cM (Z = 78) by genotyping an additional 359 offspring for 94 microsatellites present or newly generated within the first defined interval. Comparative mapping pointed out a candidate gene in this 700 kb region, namely *LIPH* (Lipase Member H). In humans, several mutations in this major gene cause alopecia, hair loss phenotypes. The rabbit gene structure was established and a deletion of a single nucleotide was found in *LIPH* exon 9 of rex rabbits (1362delA). This mutation results in a frameshift and introduces a premature stop codon potentially shortening the protein by 19 amino acids. The association between this deletion and the rex phenotype was complete, as determined by its presence in our rabbit families and among a panel of 60 rex and its absence in all 60 non-rex rabbits. This strongly suggests that this deletion, in a homozygous state, is responsible for the rex phenotype in rabbits.

## Introduction

Rex fur in animals encompasses different abnormal hair coat phenotypes due to spontaneous or induced mutations. Rex coat mutations were described in cats [1],[2], rats [3],[4], mice [5] (several mutations), and rabbits [6]. This heterogeneity in phenotype description, even within species, suggests that different genes and mutations are responsible for these phenotypes. We were interested in deciphering the molecular basis of this trait in strains of rex rabbits bred in our institute for both scientific and commercial purposes. Rabbit (*Oryctolagus cuniculus*) fur is composed of three different types of hairs: guard hair (3–4 cm long for a diameter of 50–60 µm), awn hair (3–3.5 cm/25–30 µm) and down hair (2.5–3 cm/15 µm). Guard hair and awn hair constitute the physical protection usually called the outer coat, while down hair ensures the thermal protection of the rabbit named the inner coat. Down hair is the most abundant and represents about 90–95% of all hairs. There is variability in the ratio number of the inner and the outer coat hairs, and professionals are looking for a reduction of the coarse hair quantity in rex rabbit furs.

**Figure 1:**
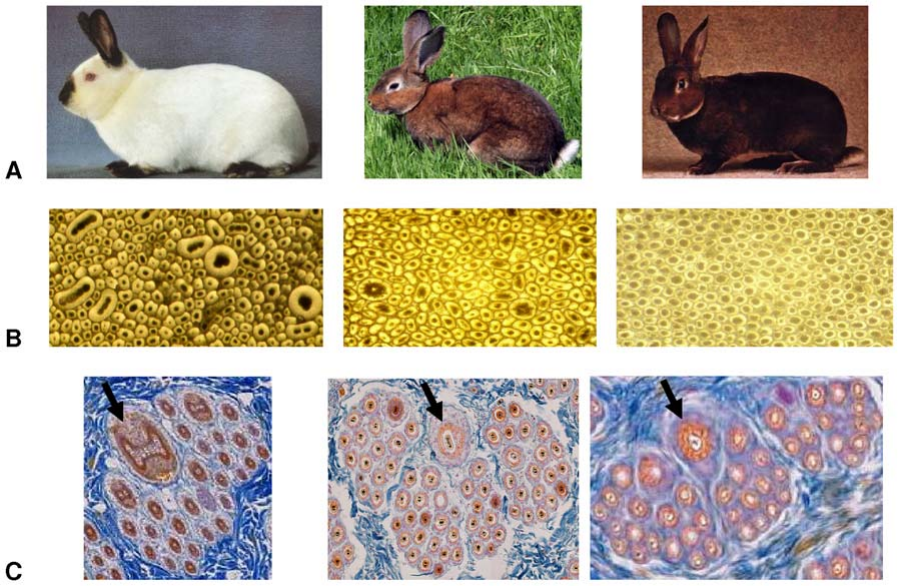
The rex hair trait in rabbit. (A) A normal rabbit coat (A1), a rex rabbit with a castor coat (A2) and an *orylag*® with a castor coat (A3). (B) Cross section of a fibre bundle of a normal rabbit coat (B1), a rex rabbit (B2) and an *orylag*® (B3). (C) Skin cross section of a hair follicle group of a normal rabbit coat (C1), a rex rabbit (C2) and an *orylag*® (C3). Arrows indicate the differences in shape and diameters of primary central hair follicles and their hair.

Hair is produced by hair follicles located in the dermis but made of epidermal cells. There is one hair synthesized per follicle and the type of hair depends on the type of follicle. In rabbits, hair follicles are structured into groups: a group is usually constituted of one central primary hair follicle surrounded by 2–4 lateral primary hair follicles and by 20–50 secondary down hair follicles (Figure 1). These three types of hair follicles appear sequentially during fetal development and early after birth. At day 19 of gestation, the central primary hair follicles increase followed at day 25 by the primary side hair follicles. At day 29 of gestation a secondary hair follicle appears for each of the 2 to 4 lateral hair follicles. Finally, secondary derived hair follicles, emerging from the skin by the same hair channel, appear during the early childhood of the animals [7]. In 1919 in the Pays de Loire French region, a mutant phenotype with soft hair was observed by a breeder in a litter of wild gray rabbits. Abbé Gillet, a local priest, considered the trait very fancy and started planning crosses to produce rabbits with this soft hair touching phenotype with guard hair that was no longer than the undercoat. In Germany in 1926 similar, if not identical, phenotypes appeared and the year after in France in Himalayan rabbits. These three phenotypes show simple Mendelian recessive inheritance likely being controlled by three different loci r1, r2 and r3 [8]. This abnormally short hair trait is thought to originate from the degenerescence of primary hair follicles leading to lacking or shortening of guard hair [9]. Interestingly, the whiskers (vibrissae) of the rex animals are bent or curly.

In the early nineteen-eighties, the trait was selected at the INRA experimental farm of Le Magneraud to further reduce the number of guard and awn hairs and is now commercialized as *orylag*® for fur and “Rex du Poitou” for its high quality meat, conferring these animals a high value. In addition, segregation of the rex hair trait was studied in experimental families (G2) using rex and common type rabbit strains (G0). Previous results suggest that these rex experimental rabbits carried only the r1 mutation and that the determinism of this trait is monogenic, autosomal and recessive.

In this study we used a positional cloning approach to identify the gene and the causal mutation of the r1 rex hair trait to help breed rex rabbits and to better understand hair growth and its underlying metabolic pathways.

## Results

### Phenotype

At first, the phenotype of the rex and normal rabbit coat was determined by visual assessment of the presence of curled whiskers associated with the content and length of coarse fibre. Then fine phenotyping of rex and normal coats was undertaken. Further analysis included objective measurements of the length of both the outer and the inner coat, fibre diameter and coarse fibre content were performed on 44 rex and 54 and normal G2 rabbits.

There was no significant difference in fibre length between the outer and the inner coat in rex individuals (W = 1154; p = 0.1149) while in normal coat rabbits the outer coat is 9.8 mm longer than the inner coat (W = 1666; p = 5.2 10^−9^). The average length of both the outer coat and the inner coat is shorter than in normal rabbits (H = 72.53, p = 1.64 10^−17^ and H = 62.95, p = 2.12 10^−15^).

**Figure 2:**
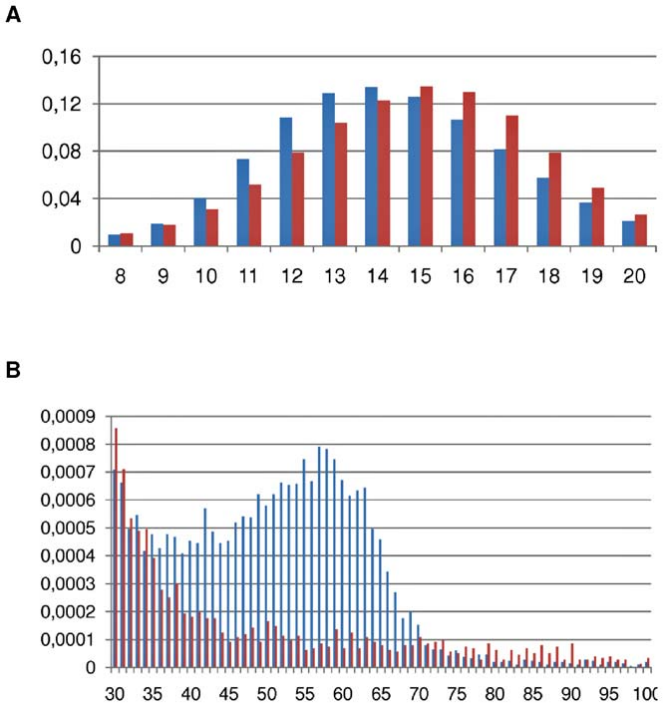
Distribution of hair diameters in µm (X-axis) in normal (blue) and rex (red) rabbit coats. Two diameter sizes ranges are represented on two different graphs to cope with the Y-axis scale (frequencies).

The distribution of hair diameter is bimodal in normal coat rabbits, the first peak corresponding to down hair ([6 µm–20 µm] centered on 14 µm with W = 0.90; p = 0.101), the second to guard hair ([45 µm–65 µm] centered on 55 µm with W = 0.96; p = 0.48). Awn hairs are less abundant and are distributed in between. The situation is quite different in rex animals especially concerning coarse hair. The down hair category (< = 20 µm) also shows a normal distribution as in normal coat but is shifted towards a larger diameter in the rex animals (Figure 2A). The second peak is not detectable due to a decrease in the number of large diameter hairs (Figure 2B).

Assuming that 30 µm is the value of hair diameters above which humans feel a fur hard at touching, coats of non rex rabbits display a higher coarse fibre content (2.28%) than rex fur (0.96%). In addition, a larger number of secondary hair follicles were observed in rex than in non-rex rabbits by histology on 20 normal and 20 rex skin cross sections (Figure 1C) in accordance with the microscopic observation of fibre bundles (Figure 1B). We also observed that all primary hair follicles from rex rabbits were present in the follicular groups and each of these hair follicles produced a hair with circular shape like downs (Figure 1C).There were very highly significant differences (p<0.001) in the diameter and length of fibers as well as the coarse fiber content between the two coat phenotypes (Table 1).

**Table 1:** Mean length (± standard deviation) of outer coat, inner coat, fibre diameter and coarse fibre content (defined as the content of fibres having a fibre diameter larger than 30 µm) in rex and normal G2 rabbit coats.

		Presence	Length of	Length of	Diameter of inner	Diameter of outer coat	
		of curled	outer coat	inner coat	coat «downs»	«awns»	«guards»
	n	whiskers	(mm)	(mm)	[0 µm; 20 µm]	[20 µm; 45 µm]	>45 µm
Normal coat	54	no	33.4±3.3	23.6±2.1	93.1%	5.4%	1.5%
Rex coat	44	yes	18.9±1.9	95.9%	3.7%	0.4%	

Finally, this study shows that the percentage of down hairs (< = 20 µm), awns ([20 µm; 45 µm]) and guard hairs (>45 µm) fits with the expected distribution of a follicular group in the common rabbit, one primary central, 2 to 4 primary lateral and 30 to 50 secondary follicles.

### Mapping of the rex gene

#### Genome scan

The segregation of the rex trait in the G2 offspring confirmed an autosomal recessive determinism of this character. A whole genome scan was performed with 109 microsatellite markers from the first genetic map on rabbit families in which the rex trait was segregating [10]. Linkage analysis mapped the rex phenotype on chromosome 14 within an interval of 40 cM.

#### Fine mapping

In order to reduce the mapping interval of the trait, we produced and characterized microsatellites within the primo-localization region by two methods. First, we produced 19 of these polymorphic markers by screening the rabbit Bacterial Artificial Chromosome (BAC) library [11] for 14 Expressed Sequenced Tags (EST) potentially localized in the 40 cM interval and selected by comparative mapping with the homologous human chromosome region (HSA3). We also retrieved 152 microsatellite markers with a dinucleotide motif of at least 18 repetitions and well distributed in this HSA3 region by *in silico* screening. Forty-eight of these were selected and polymorphism was tested on a multi-origin rabbit panel and 23 were found segregating in our rabbit families. In total, we produced 171 markers in the first 40 cM interval and 42 were finally genotyped on our families for linkage analysis (19 from BAC library screening and 23 retrieved *in silico*).

**Figure 3:**
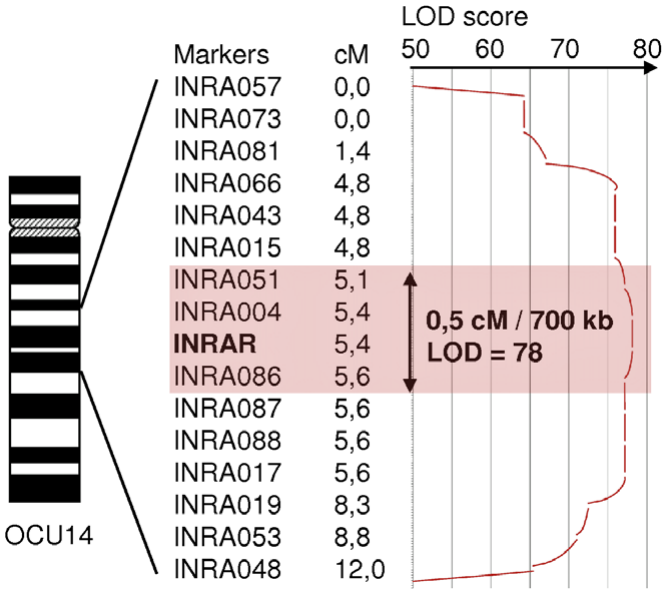
Mapping of the rex coat trait in the rabbit families. A whole genome scan performed on 187 rabbits for 47 microsatellites localized the rex phenotype (INRAR) on rabbit chromosome 14 within an interval of 40 cM. Fine mapping refined the localization within a 0.5 cM (⊖ = 0 ; LOD = 78) region flanked by microsatellites INRA051and INRA086.

Microsatellite INRA004 shows the most significant linkage with the rex phenotype among the 359 rabbits (LOD = 78; ⊖ = 0). The final interval of localization for rex was estimated at 0.5 cM (700 kb) flanked by INRA051 (LOD = 31; ⊖ = 0.03) and INRA086 (LOD = 40; ⊖ = 0.01) markers. INRA086 belongs to a 4 marker linkage group (INRA086-087-088 and INRA017) with no recombinant and was arbitrarily considered as the lower limit of the interval (Figure 3).

### Candidate Gene

In the last 0.5 cM interval, we identified three genes by comparative mapping of this region with mouse and human sequences since no appropriate rabbit sequence was available. Screening the rabbit BAC library identified a BAC sequence containing INRA017 and a part of the *SENP2* gene (first gene upstream of the last interval) and a BAC containing the INRA004 and three genes: *MAP3K13*, *TMEM41A* and *LIPH*. *MAP3K13* is a member of the serine/threonine protein kinase family which could play a role in the *JNK* (c-Jun NH(2)-terminal kinase) signaling pathway. *TMEM41A* is a transmembrane protein of unknown function and not yet characterized. *LIPH* is a membrane-bound member of the mammalian triglyceride lipase family, the phosphatidic acid-selective phospholipase A1 (PLA1). It specifically hydrolyzes phosphatidic acid (PA) to produce 2-acyl lysophosphatidic acid (LPA), which is a lipid mediator with diverse biological properties that include platelet aggregation, smooth muscle contraction, and stimulation of cell proliferation and motility. Disruption of the *LIPH* gene in the mouse results in various phenotypes including retarded hair growth and postnatal lethality [12]. Moreover, numerous mutations have been found in the *LIPH* gene. In 2006, the deletion of the region encompassing exon 4 was identified as being responsible for Hypotrichosis Simplex (HS) in 50 families of Russian people [13]. The Hair of these patients is abnormally short, dystrophic and fragile due to retarded or arrested hair growth. Mutations have also been reported in exon 1 [14], exon 2 [14]–[18],[21], exon 4 [21], exon 5 [17],[19]–[22], exon 6 [23],[24], exon 7–8 [21] and in exon 9 [22]. *LIPH* was thus considered as a strong candidate gene for the rex phenotype.

### Tissue expression

**Figure 4:**
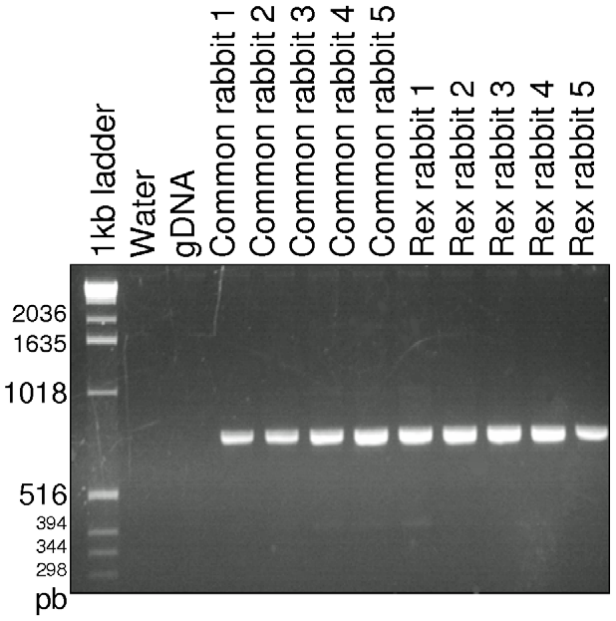
LIPH expression in rabbit skin. LIPH was detected by RT-PCR (ex 7–10) in rabbit skin of 5 common and 5 rex rabbits. The expected product size is 753 pb.

The expression of *LIPH* was detected in the intestines, muscles, kidney, lung, spleen and liver by RT-PCR (data not shown) and in the skin from the adult wild type and rex rabbits as shown in Figure 4. No alternative transcript was detected in the skin using the four primers covering the coding area (Table 2, Figure 4).

**Table 2:** Primers used for RT–PCR.

	cDNA primers	Amplicon size (in pb)
**ex1-4**	F1_AACCAGGGGAAACCTAAAGC	728
	R1_TCAGTGTCGGAATGGATGAC	
**ex2-8**	F2_CCAGTTTGGTTGCAGGACTT	889
	R2_TGGATTCTGTGGTGTTTCCA	
**ex7-10**	F3_TGAGAAAGACCCTCCAATGA	753
	R3_TGCTGATGGACAGCAGAATC	
**ex9-10**	F4_TCATCAAGTGAGTCTGCTTGC	781
	R4_CACAGATGTGACACCCATGA	

### Gene structure

**Figure 5:**
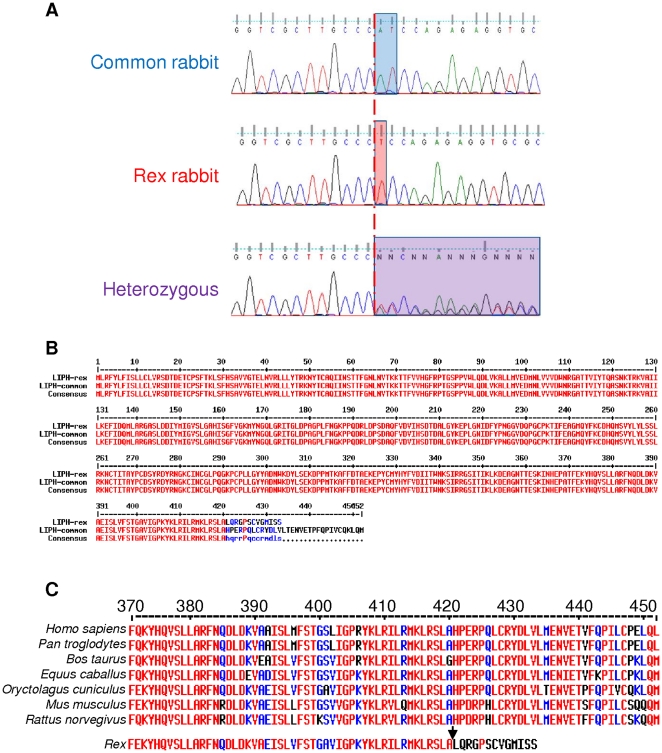
The 1362delA mutation in exon 9 of LIPH in rex rabbits. (A) Electropherograms of the LIPH exon 9 sequence from a normal common type rabbit (WT), a rex and a heterozygous are shown. The red line indicates the location of the mutation. In heterozygous rabbits there is an overlap of both allele sequences (purple box). (B) Deduced alignment of LIPH proteins between rex and normal common type rabbits. (C) C-terminal conservation in mammalians.

Genomic DNA and messenger RNA sequencing allowed us to determine the *LIPH* gene structure in the rabbit. The gene is composed of 10 exons as in other species. The length of the open reading frame is 1932 pb with a predicted protein of 452 amino acids (Figure 5B).

Sequence analysis of this rabbit cDNA shows an identity of 90% compared to other mammalian species. The *LIPH* protein sequence is also highly conserved along mammalians with more than 80% of identity, including the catalytic domain and the C-terminal region (Figure 5C). We noticed that the rabbit *LIPH* lid domain is one amino acid longer (Glu at position 239) than that of the other species.

### Mutation screening and distribution

By sequencing the complete coding region of the *LIPH* gene in 4 rex and 4 non rex rabbits, we identified a deletion of one nucleotide (1362delA) located in exon 9 and present in a homozygous state in the rex rabbit gene sequence (Figure 5A). This mutation leads to a frameshift and the appearance of a premature stop codon 32 amino acid downstream potentially resulting in a 19 amino acid truncated protein (Figure 5B). No other sequence differences were detected in the nine other exons of the gene.

The distribution of the 1362delA allele was firstly investigated among rabbits used for the mapping. The 8 G1 and 66 G2 common type rabbits were all heterozygous as expected, and the 8 G1 and 81 G2 rex rabbits were homozygous for the deletion. To confirm this result, the deletion distribution was investigated among unrelated rex animals and strains of standard rabbits. All 60 rex rabbits were tested homozygous for the 1362delA while all 60 rabbits that did not have the rex phenotype were tested homozygous for the wild allele.

## Discussion

Macroscopic visual assessment for the presence of curled whiskers, length and content of coarse fibres is efficient to categorize rabbits having a typical defined phenotype in rex or normal coat type as proposed by Castle [8]. However due to variability amongst individuals, it is difficult to classify with certainty few animals having an intermediate coat. Objective measurements of the length of both the inner and the outer coat and coarse fiber content were found to be very helpful. Fine phenotyping using a combination of several criteria including, the length of both guard hair and down, the difference in length between outer and inner coat and measure of fiber diameter distribution greatly improved the classification of the animals. It allowed an accurate description of the rex phenotype in rabbits and was a necessary step to undertake for the positional cloning approach.

Rex rabbit guard hair and down were shorter than in the normal rabbit coat which was in agreement with earlier observations [6],[9]. The decrease in the length of guard hair (−43%) was double than that observed (−20%) in down leading to a closely similar length of both guard hair and down in rex rabbits. This situation can explain the often erroneous description of lacking guard hair in the rex rabbit due to a simple macroscopic view of the fur.

A decrease in hair length could be due to either a shorter duration of hair growth or a slower hair growth rate. By comparing rex and normal rabbit coats, Vrillon [9] did not observe any differences in the duration of the moulting process indicating that hair growth duration was similar in both genotypes and thus strengthening the hypothesis that a decrease in hair length could be due to a decrease in hair growth rate in agreement with observations in humans [13].

Histology allowed us to state that the lack of guard hair in rex rabbits was not due to the degenerescence of primary hair follicles. It also highlighted that primary hair follicles in rex were still active and produce hair. These guard hairs present a modification of their shape and an important decrease of diameter. The hair produced by the primary follicles seem to have disappeared but their reduction in size and shape, especially for guards, made them look like down. This is the reason why guard and awn hairs have been considered as lacking when fur was only visually assessed. It also explains why rex fur is soft touching, plush-like and represents a great value for breeders and the industry.

A positional cloning approach was undertaken in rabbit families segregating for the rex phenotype and for microsatellite markers. Linkage analysis after whole genome scanning and fine mapping allowed us to localize the rex coat trait in a 0.5 cM region on OCU14. In this 700 kb interval, comparative mapping with the human and mouse sequences pointed out *LIPH* as a strong candidate gene.

A *LIPH* ^lpd1^ Knock Out mouse exhibited a complex multifactorial phenotype including retarded hair growth [12]. In humans, mutations in a small number of genes have been recently identified for a group of alopecia called Hypotrichosis Simplex (HS). Alopecia is characterized by patchy hair loss on the scalp that can eventually involve the entire scalp (*alopecia totalis*) or the entire body (*alopecia universalis*) [25]. Autosomal recessive hypotrichosis is characterized by sparse scalp hair and fragile hair. Three autosomal recessive forms *LAH1*, *LAH2* and *LAH3* have been found associated with three different genes, *DSG4* [26], *LIPH* [13] and *P2RY5* [27] respectively. Several lanes of evidence were in favour of the *LIPH* gene. We have shown by RT-PCR that *LIPH* was expressed in rabbit skin. Then, we established the *LIPH* gene structure by sequencing all its exons. The normal *LIPH* mRNA sequence presented in this study differs from that found in rabbit lachrymal glands by 5 nucleotide substitutions, one of which introduces an amino acid change (p.Ala369Val) [28].

A single polymorphism was found as a one base pair deletion in exon 9 (1362delA) of *LIPH* when comparing sequences from rabbits of normal wild and rex phenotypes. This deletion most probably leads to a premature termination of the rex *LIPH* protein. Segregation analysis of the 1362delA showed a complete linkage with the rex phenotype across families used for mapping and a total association with the trait on a 120 rabbit panel confirming the recessive determinism of the trait.

*LIPH* is a membrane-bound member belonging to the mammalian triglyceride lipase family. This gene has a phospholipase A1 activity that catalyses the production of 2-acyl LysoPhosphatidic Acid (LPA) by hydrolyzing Phosphatidic Acid [29]. LPA is a lipid mediator whose biological properties include platelet aggregation, smooth muscle contraction, and stimulation of cell proliferation and motility.

Hair formation is the result of the differentiation of keratinocytes during their migration along the hair shaft. Since *LIPH* encodes PA-PLA1α with a pla1 activity expressed in the skin, it has been speculated that loss-of-function of *LIPH* would reduce the production of LPA in hair follicles that could affect the migration, differentiation, or proliferation of keratinocytes, culminating in the arrest of hair growth [13]. PA-PLA 1α has three amino acid residues in the N-terminal domain, Ser154, Asp178 and His248, which form the putative catalytic triad [29]–[32], a β9 loop and a short lid domain which are considered as the structure involved in substrate recognition [29],[33],[34]. All the homozygous mutations in *LIPH* found in humans directly impact the lipase domain of the PA-PLA1α or lead to a premature termination codon (PTC).

In rex animals, the 1362delA mutation in *LIPH* affects the C-terminal of PA-PLA1α. Since the protein is localized to the plasma membrane [35], we hypothesize that the C-terminal region is involved in the final localization of *LIPH* where it is active. No specific amino acid sequence pattern was found in this region, but it is noticeable that the C-terminal region is highly conserved in mammalians. Moreover, in humans, a compound mutation including one in the C-terminal has been found leading to HS [22]. The phenotype was that of classic HS suggesting a role of this domain in the PA-PLA1α protein function. Rex rabbits show the first mutation in the C-terminal of PA-PLA1α in a homozygous state involved in a hair growth defect. To that point, it is not possible to exclude other hypotheses such as nonsense mediated decay (NMD) in which most likely aberrant transcripts with a PTC would be largely degraded. The 1362delA mutation could also affect the tertiary structure of PA-PLA1α and then reduce or destroy its activity.

The *LIPH* mutation found in this study to be associated with the rex coat trait confirms the importance of this gene in the hair follicle metabolism in mammals. Contrarily to the situation encountered in humans, this deletion in rabbits does not lead to sparse hair and is considered as an advantage for professionals. A molecular test is already available for breeders as a tool for improving their breeding programs or for introgression of the rex gene in any other rabbit population.

It is the first time that a mutation in the C-terminal carried in the homozygous state is shown to provoke a short hair phenotype, highlighting a possible important role of this domain in the *LIPH* function. Further studies are needed to define the role of this mutation in the dysfunction of the *LIPH* protein and its effect on hair growth retardation.

## Materials and Methods

### Rabbit families and reference population

#### Mating plan

Three rabbit INRA strains were used to build three generation families. Two crosses were carried out to produce the G1 generation : (i) eight INRA 2066 wild type (RR) males with 22 rex does (rr) and (ii) three rex males (rr) with 12 rex does (rr). The 53 G1 does (Rr) from the (i) cross were then mated with the 29 G1 males (rr) from the (ii) cross, which produced 853 G2 rabbits with theoretically 50% of homozygous rex (rr) and 50% of heterozygous showing wild phenotype (Rr). All rex rabbits used in this study come from the *orylag*® strain.

#### Phenotyping rabbits

Rabbits were phenotyped for coat texture. All G2 rabbits were firstly examined macroscopically by visual assessment for the presence of curled whiskers, the length and the content of coarse fibres. At first, a visual macroscopic observation was considered efficient to categorize rabbits with rex or normal coat type. To improve accuracy, a fleece sample was taken on all animals for objective measurements of the length of the outer and the inner coat with a rule. Finally, a subset of 44 rex and 54 normal coat G2 rabbits were further analyzed for mean fibre diameter, fibre distribution and content of coarse fibres. The latter was defined as the percentage of fibres having a diameter larger than 30 µm by measuring 4000 individual fibre snippets per sample using the Optical Fibre Diameter Analyzer (OFDA) methodology as previously described [36]. Royston's sign for the Shapiro-Wilk test was used to assess the normality of the hair distributions. Kruskall Wallis was then used to determine the difference between normal coat and rex rabbit hairs and inner/outer for both phenotypes.

#### Blood collection and DNA extraction

Eight milliliters of blood were sampled in vacutainers (K3-EDTA; Becton Dickinson, Rutherford, USA) by cardiac puncture on all rabbits. DNA was extracted from peripheral blood mononuclear cells as described by Jeanpierre [37]. This experiment was licensed under the guidelines of the French Ministry of Agriculture and in agreement with the rules **of** the National Committee of Animal Experimentation for animal research. Blood samples were drawn by authorized skilled staff in our Inra experimental station of Le Magneraud and all dispositions were taken to minimize suffering (authorization certificate to experiment on living animals N°78–103).

In France, no mandatory ethical committee approval is yet necessary to conduct experiments on the animals of this study.

### Mapping of the rex gene

#### Primo localization

A genome scan was achieved by genotyping 187 G2 rabbits for the 109 microsatellite markers of the genetic rabbit map [10]. A low-cost technique described by Schuelke (2000) was applied for the genotyping of microsatellites. The PCR reaction was carried out with three primers, including a locus specific primer extended with a universal sequence of 17 nucleotides (5′- GACCGGCAGCAAAATTG-3′), a reverse locus specific primer and a universal primer of 17 nucleotides that was 6-Fam, Hex or Tet 5′-labelled (MWG AG Biotech, Ebersberg, Germany). The results were analysed with the Genetic Profilerv1.1 software (ABIPrism377A sequencer; Applied Biosystems, Foster City, CA, USA) or the Genotyper software (MegaBACE1000 sequencer; Amersham Biosciences, New Haven, CT, USA). Genotyping data were checked using an in-house program, which analyzed the consistency of the allele distribution of each marker according to pedigrees.

The CRI-MAP 2.4 software (Green et al., 1990) was used to build the genetic map. The first step identified linked markers by a two-point analysis with the two-point option. In the second step, the linkage groups were examined by multipoint analysis using the build and flipsn options. Linkage data were merged with cytogenetic mapping data to confirm the order of markers or to identify linkage of isolated or weakly informative markers. These various analyses were performed with a LOD score of 3 (lowered to 1.8 in a few cases where the cytogenetic position of the markers made it possible to confirm the proximity). The size of the genetic map was calculated by adding up the genetic distances of all linkage groups plus 15 cM at both ends of each group and including 15 cM on both sides of each unlinked marker.

#### Fine Mapping

Three-hundred fifty-nine rabbits belonging to a total of 17 families were genotyped with an additional 42 microsatellites. The CRI-MAP software version 2.4 [38] was used to construct the map. We used MultiMap to generate maps and calculate the probabilities for orders obtained from an initial order of reference, by permutation of n adjacent loci.

#### Characterization of microsatellite markers

Microsatellite markers localized in the primo localization area were produced by molecular genetic approaches in BAC as described by Chantry-Darmon [39].

An *in silico* approach was also used. The rabbit sequences OryCun1 (2× low-coverage assembly, 777141 sequences in Ensembl comprising traces, EST, contigs, scaffolds and not located on the genome) were compared by BLAST with the human masked sequences of chromosome 3 for the 170 Mb–190 Mb interval of primary location (OCU 14q17). Only sequences with a significant BLAST score set with an “e” value greater than or equal to 10^−40^ were kept. Microsatellite identification in these sequences has been implemented using Tandem Repeat Finder [40]. Finally, primers to amplify the microsatellites were designed using Primer3 [41]. Three zones of fragment sizes were chosen to allow multiplexing: 100±20 bp, 150±20 and 200±20 bp. We selected uninterrupted microsatellites with at least 18 repetitions in order to improve the probability of observing polymorphism.

#### Linkage analysis

We used Simwalk2 version 2.91 [42] to perform linkage analysis based on the Z score method taking the family structure, the number of individuals and markers into account. A parametric test assuming a recessive model with complete penetrance and a nonparametric test, with the respective options “parametric linkage analysis” and “non-parametric linkage (NPL) analysis” were used.

Haplotype analysis was also conducted with Simwalk2 v 2.91 and the results were analysed using Haplopainter version 27beta [43] and Ghostscript 8.54.

#### Gene structure analysis

Total RNA were extracted from different tissues using the RNA Now procedure (Biogentex). Reverse transcriptions (RT) were performed on 5 µg of total RNA using the Superscript First Strand Synthesis System (Invitrogen) following the manufacturer's instructions. RT on rabbit RNA were performed using oligonucleotides(dT)18 followed by PCR using the GoTaq® Flexi DNA Polymerase and reaction buffer (Promega).

The cDNA sequencing of rabbit *LIPH* was performed after RT-PCR amplification using 4 primer pairs designed to amplify overlapping fragments 500 à 600 bp long covering all the exons of the genes (Table 2). All oligonocleotides were designed using the primer 3 software (http://frodo.wi.mit.edu/primer3/). The PCR amplification reactions were optimized and carried out in a PTC-100 (MJ-Research) using the following cycling conditions: 95°C for 5 min followed by 35 cycles of (95°C for 30 s, annealing temperatures 60°C for 30 s, 72°C for 30 s) and 72°C for 5 min. The resulting PCR products were separated on a 2% agarose gel, purified using Wizard SV Gel and PCR Clean-Up System (Promega, Wisconsin, USA) and sequenced by Qiagen sequencing services. The sequences were assembled in clusters of contiguous sequences using the CAP3 assembler [44] using default parameters.

The resulting sequences were compared to the rabbit and human genome sequences in the NCBI database by means of the BLASTN software (http://www.ncbi.nlm.nih.gov) to deduce the intron/exon structure of the *LIPH* gene. All *LIPH* genomic and cDNA sequences generated during the course of this study have been submitted to GenBank databases (HQ845290).

#### *LIPH* Polymorphism

All exons of the *LIPH* gene were amplified using primers in the introns and both sides of the exons for 4 rex and 4 common rabbits. The PCR were performed on genomic DNA extracted from blood of our rabbit families. PCR reactions were optimized using the following cycling conditions: 95°C for 5 min followed by 35 cycles of (95°C for 30 s, annealing temperatures (Table 3) for 30 s, 72°C for 30 s) and 72°C for 5 min. DNA fragments were then analyzed for the gene structure analysis as described above. Primers used are listed in Table 1. The resulting sequences were introduced into the NovoSNP software [45] to find Single Nucleotide Polymorphism in the amplified sequences and compared using “multalign” [46] to find any other polymorphism.

**Table 3:** Primers used for genomic amplification.

	Exon size	gDNA primers	Amplicon size	Annealing temperature
	(in bp)		(in bp)	(in °C)
**Exon 1**	149	F1_GCCAGGCACCATTCTAAAAG	345	60°C
		R1_TGAAAAGAGGAGGAGCCAAA		
**Exon 2**	368	F2_TGGGGCTTATTCAGATTTGC	574	60°C
		R2_TGCAGTGAATAGCAGAGGATTC		
**Exon 3**	109	F3_CAGCTAGGACACTTCTCCAAA	292	60°C
		R3_TGGAAAAGCTGGCTTTGAAC		
**Exon 4**	102	F4_ACGAACCATCGAATCAGGAA	267	60°C
		R4_GCTACCCCCAGGGAGAGACT		
**Exon 5**	90	F5_GCTCCCTCTCTCGCTGTAAC	400	60°C
		R5_CGCAGCTAACTATTGGGGTAA		
**Exon 6**	171	F6_AGGGAAACTGCTTGTTGGAC	208	58°C
		R6_CTCTACAAAGCCAGGGATGC		
**Exon 7**	96	F7_AGAAGTGGCTGGGAACCTG	262	60°C
		R7_CCAATGCGCTGTTCTCATTA		
**Exon 8**	112	F8_AGAGCAGAAGTGCAGAACCA	299	60°C
		R8_GTTATCAGGGGGATGGGTTT		
**Exon 9**	174	F9_TCTCCCTGACTTTTCCTACTTCA	298	58°C
		R9_CCCTCCCCAAATAAATCTTTTAAC		
**Exon 10**	607	F10_ACACTGCAGAGAAGGCAGGT	836	60°C
		R10_AGCGTGGCTCCTGTTCATTA		

#### Association study

A first panel of rabbits used for mapping was genotyped by direct sequencing for the deletion using primers F9/R9 (Table 3). This panel was constituted by 8 G1 normal and 8 G1 *orylag*® rex-rabbits and 66 G2 wild type rabbits and 81 G2 *orylag*® rex-rabbits. A panel of 60 common rabbits and 60 unrelated *orylag*® rex-rabbits was then genotyped.
